# Multiscale Nest-Site Selection of Burrowing Owl (*Athene cunicularia*) in Chihuahuan Desert Grasslands

**DOI:** 10.3390/biology15030236

**Published:** 2026-01-27

**Authors:** Gabriel Ruiz Aymá, Alina Olalla Kerstupp, Mayra A. Gómez Govea, Antonio Guzmán Velasco, José I. González Rojas

**Affiliations:** 1 Universidad Autónoma de Nuevo León (UANL), Facultad de Ciencias Biológicas, Laboratorio de Biología de la Conservación y Desarrollo Sustentable, Cd. Universitaria, San Nicolás de los Garza 66455, Nuevo León, Mexico; gabriel.ruizym@uanl.edu.mx (G.R.A.); alina.olallakrs@uanl.edu.mx (A.O.K.); mayra.gomezgv@uanl.edu.mx (M.A.G.G.); 2 Universidad Autónoma de Nuevo León (UANL), Facultad de Ciencias Biológicas, Laboratorio de Ornitología, Cd. Universitaria, San Nicolás de los Garza 66455, Nuevo León, Mexico

**Keywords:** burrowing owl, nest-site selection, multiscale habitat selection, grassland, Chihuahuan desert, logistic regression

## Abstract

Nest-site selection in birds is a hierarchical process shaped by environmental filters operating across multiple spatial scales. In species that depend on burrows excavated by ecosystem engineers, understanding how these filters interact is essential for effective conservation. We evaluated nest-site selection by the Burrowing owl (*Athene cunicularia*) within colonies of the Mexican prairie dog (*Cynomys mexicanus*) in the southern Chihuahuan Desert using a multiscale analytical framework spanning burrow, site, colony, and landscape levels. During the 2010 and 2011 breeding seasons, we located 56 successful nests and paired each with an inactive non-nest burrow within the same colony. Eighteen structural and environmental variables were measured and analyzed using binary logistic regression models, with model selection based on an information-theoretic approach (AICc) and prior screening for predictor collinearity. Nest-site selection was associated with greater internal burrow development and reduced external exposure at the burrow scale, proximity to satellite burrows and low-to-moderate vegetation structure at the site scale, higher densities of active prairie dog burrows at the colony scale, and reduced predation risk and agricultural disturbance at the landscape scale. The integrated multiscale model showed substantially greater support and discriminatory power than single-scale models, indicating that nest-site selection emerges from interactions among spatial scales rather than from isolated factors. These findings support hierarchical habitat-selection theory and underscore the importance of conserving functional Mexican prairie dog colonies and low-disturbance grassland landscapes to maintain suitable breeding habitats for Burrowing owls in the southern Chihuahuan Desert.

## Simple Summary

Burrowing owls depend on underground burrows to reproduce, but these burrows are not consistently available across landscapes. In northern Mexico, Burrowing owls rely almost entirely on burrows created by Mexican prairie dogs, an endemic species currently at risk of extinction due to habitat loss. Understanding how owls choose where to nest is essential for protecting both species and the grasslands they inhabit. We examined how Burrowing owls select nesting sites across spatial scales, from the characteristics of individual burrows to broader landscape features. We found that owls prefer burrows that offer greater internal space and protection, are located near other available burrows, occur in prairie dog colonies with moderate activity, and are found in landscapes with fewer structures that predators can use and farther from croplands. Our results show that nesting decisions are shaped by a combination of local and landscape conditions rather than by a single factor. These findings highlight the importance of conserving functional prairie dog colonies and maintaining low levels of human disturbance in grassland ecosystems. Protecting these conditions will help ensure suitable breeding habitat for Burrowing owls and support the conservation of grassland biodiversity in northern Mexico.

## 1. Introduction

Habitat selection in birds is a hierarchical process in which individuals choose among available alternatives across multiple spatial scales [1,2]. This process integrates innate and learned decisions, ranging from macrohabitat selection to specific microhabitat attributes [3,4,5]. Within this framework, nest-site selection is a critical stage of habitat selection because it directly affects the survival of eggs, nestlings, and breeding adults. Factors such as vegetation structure and composition strongly influence food availability, perch presence, protection from predators, and the suitability of nesting sites [6,7,8].

The Burrowing owl (*Athene cunicularia*) is a diurnal, fossorial species that does not excavate its own burrows and therefore depends on abandoned burrows created by a variety of burrowing mammals [9,10,11]. In North America, this species shows a strong association with colonies of the Black-tailed prairie dog (*Cynomys ludovicianus*), a relationship documented in both Canada and the United States [9,12,13,14]. In Mexico, Burrowing owls primarily occupy burrows of the Black-tailed prairie dog and the endemic Mexican prairie dog (*C. mexicanus*) [14,15,16,17,18].

The Mexican prairie dog, restricted to portions of Coahuila, Nuevo León, San Luis Potosí, and Zacatecas, is listed as an endangered species under Mexican law [19] and plays a fundamental ecological role in the arid grasslands of northeastern Mexico. Its colonies increase habitat heterogeneity and biological diversity and provide refuges used by numerous faunal groups, including predators that prey on prairie dogs [20]. For the Burrowing owl, classified as a species under special protection in Mexico [19] and considered threatened in Canada and the United States [21,22], the availability and quality of functional burrows are a key limiting resource essential for completing its reproductive cycle.

Anthropogenic degradation of grasslands from agricultural, livestock, and urban expansion has reduced the continuity of prairie dog colony complexes, directly affecting the availability of suitable burrows and, consequently, the reproductive success of Burrowing owls [17]. Nesting sites are particularly vulnerable to both natural and anthropogenic disturbances, which alter the relative availability of suitable sites and modify habitat-selection patterns across spatial scales [23].

Nest-site selection by the Burrowing owl is influenced by a set of physical and environmental attributes, with the availability of suitable burrows being the critical factor [24,25]. These burrows must meet specific structural criteria, including entrance diameter, depth, length, and orientation, as documented for populations in the United States [26,27,28]. In addition, surrounding microhabitat conditions, particularly vegetation density and composition, influence thermoregulation, protection from predators, and overall nest viability [14,16,17,26].

Numerous studies have shown that nest-site selection in this species is hierarchical and scale-dependent, ranging from the landscape to the individual burrow and responding to factors such as proximity to roads, colony size, the activity level of *Cynomys* spp., the density and spatial distribution of available burrows, vegetation structure, and burrow dimensions [29,30,31]. Multiscale analyses conducted in North America and South America consistently indicate that no single spatial scale fully explains burrow occupancy, emphasizing the need for integrated analytical frameworks [25,26,32]. Despite this body of knowledge, few studies in Mexico have evaluated Burrowing owl nest-site selection within Mexican prairie dog colonies using an explicit multiscale framework, even though this system is highly fragmented and of high conservation priority. This limitation constrains regional understanding of the species’ ecology and limits assessment of the transferability of multiscale models derived from temperate grassland systems to arid and disturbed landscapes. In particular, it remains unclear whether the relative hierarchy of selection filters (burrow, site, colony, and landscape) is conserved or reconfigured under conditions where burrows excavated by ecosystem engineers are spatially limited.

Based on this framework, we explicitly test the following hypothesis and associated predictions. We hypothesize that Burrowing owl nest-site selection is influenced by factors that vary across spatial scales, with occupancy probability determined by filters operating across multiple spatial scales. Under this hypothesis, structural attributes of the burrow and the immediate microhabitat are expected to exert greater influence at fine spatial scales. In contrast, colony- and landscape-level variables are expected to become more relevant at broader spatial scales. Accordingly, we predict that nests will be preferentially established in burrows with suitable dimensions and greater structural complexity, and in areas with relatively low vegetation cover and higher *Cynomys* spp. activity, reflecting non-random selection of available sites.

Nest-site selection in Burrowing owls is shaped by a combination of biotic and abiotic factors, including predator avoidance, availability of suitable burrows, and habitat structure that facilitates early predator detection. These processes operate across spatial scales, from burrow and microhabitat attributes to colony- and landscape-level features, providing the ecological rationale for the multiscale approach adopted in this study.

The objective of this study is to identify the habitat characteristics influencing Burrowing owl nest-site selection across spatial scales (burrow, site, colony, and landscape) within Mexican prairie dog colonies in northeastern Mexico. By doing so, this study aims to improve understanding of habitat-selection mechanisms in arid grassland systems and to provide information relevant for the management and conservation of both Burrowing owls and Mexican prairie dog colonies in northeastern Mexico.

## 2. Materials and Methods

### 2.1. Study Area

Sampling sites were located within the Chihuahuan Desert Ecoregion of the Mexican Plateau, encompassing portions of the states of Nuevo León, Coahuila, Zacatecas, and San Luis Potosí. The prevailing climate is semi-arid and warm, with a mean annual temperature ranging from 14 to 18 °C and an average annual precipitation of approximately 427 mm [33].

**Figure 1:**
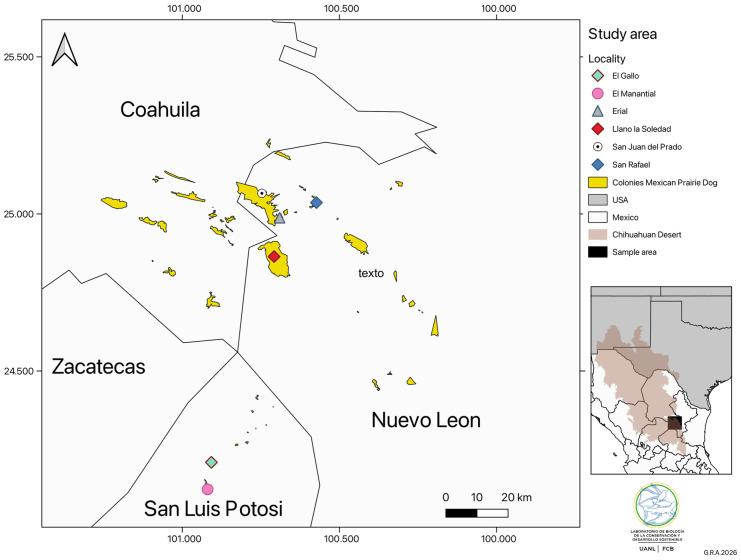
Study area in the Chihuahuan Desert and location of Burrowing owl nesting sites in the southern Chihuahuan Desert.

In Nuevo Leon, field surveys were conducted in the municipality of Galeana at the localities of El Llano de La Soledad (23°53′ N, 100°42′ W), Erial (25°00′ N, 100°40′ W), San Rafael (25°01′ N, 100°35′ W), San Juan del Prado (25°03′ N, 100°42′ W) and La Hediondilla (24°57′ N, 100°42′ W). In San Luis Potosi, sampling was conducted at Llano del Manantial (24°07′ N, 100°55′ W) and at the colonies of El Gallo “A” and “B” (24°12′ N, 100°54′ W), within the municipality of Vanegas (Figure 1). These localities form a gradient of semi-arid grasslands with varying levels of ecological integrity and anthropogenic disturbance, allowing evaluation of nest-site selection under contrasting environmental conditions.

The dominant vegetation consists of halophytic grasslands, characterized by species such as *Muhlenbergia villiflora*, *Hilaria mutica*, *Sporobolus airoides*, and *Frankenia gypsophila*, as well as microphyllous and rosette scrub communities [34,35].

Notably, El Llano de La Soledad holds multiple conservation designations, including State Natural Protected Area (ANP), Western Hemisphere Shorebird Reserve Network (WHSRN) Site of International Importance, and Important Bird Area (AICA), because it supports several vulnerable, endemic, and migratory bird species. These include Worthen’s sparrow (*Spizella wortheni*), Ferruginous hawk (*Buteo regalis*), Golden eagle (*Aquila chrysaetos*), Long-billed curlew (*Numenius americanus*), Mountain plover (*Charadrius montanus*), Sprague’s pipit (*Anthus spragueii*), and Upland sandpiper (*Bartramia longicauda*) [36,37,38]. Access to sites within the protected area and ejido lands was granted by the environmental authority and local ejidal councils.

The region has a historically high density of prairie dog colonies, which has contributed to the availability of burrows used by Burrowing owls. This spatial correspondence is particularly relevant for interpreting habitat-selection patterns within the study area.

### 2.2. Selection of Sampling Sites

#### 2.2.1. Data Collection

During March–May 2010 and 2011, an intensive search for Burrowing owl nests was conducted within Mexican prairie dog colonies, following the criteria described by [17]. Nests were identified by the presence of accumulated or modified material at burrow entrances, including cow, horse, or coyote feces, grass, paper, and plastic debris [26,39]. Once located, nests were georeferenced. Searches were conducted along linear transects 1 km in length, walked back and forth (2 linear km per locality), with a standardized sampling effort of 80 person-hours per breeding season.

Only successful nests were recorded, defined by the observation of nestlings or fledglings emerging from the burrow during June–July. This criterion was applied exclusively to ensure accurate identification of active nesting sites and to focus the analysis on nest-site selection rather than on reproductive performance. Spatial distribution maps of nest locations were generated using ArcGIS Pro (version 3.2; ESRI, Redlands, CA, USA).

#### 2.2.2. Variable Measurement Across Spatial Scales

Once burrows were identified, a total of 18 structural variables were measured and grouped into four hierarchical spatial scales (burrow, site, colony, and landscape) within a multiscale habitat-selection framework:

(a) Burrow scale: burrow type (nest/not-nest), entrance height (EH), entrance width (EW), entrance diameter (ED), burrow height above ground (BHG), distance to upper level (DUL), exit chamber length (ECL), tunnel internal diameter (TID), orientation (OR; N, S, E, W, NE, NW, SE, SW), vegetation cover (VC1 × 1), and vegetation height (VH1 × 1), measured within a 1 m^2^ area surrounding the burrow entrance. All burrow structural dimensions (EH, EW, ED, BHG, DUL, ECL, and TID) were measured in centimeters (cm). Orientation (OR) was recorded as a categorical variable corresponding to the cardinal direction of the burrow entrance (N, NE, E, SE, S, SW, W, NW). Vegetation height (VH1 × 1) was measured in centimeters (cm), and vegetation cover (VC1 × 1) was visually estimated as a percentage (%).
(b) Site scale: active burrows within 50 m (AB50) and inactive burrows within 50 m (IB50), quantified within a circular plot of 50 m radius; satellite burrow presence (SB), distance to satellite burrows (DSB), vegetation cover within 50 m (VC50), and vegetation height within 50 m (VH50). This scale represents the immediate nest surroundings, where interactions between burrow availability and microhabitat structure are integrated. Distance to satellite burrows (DSB) was measured in meters (m), vegetation height within 50 m (VH50) in centimeters (cm), and vegetation cover within 50 m (VC50) was estimated as a percentage (%).
(c) Colony scale: active burrows within 100 m (AB100) and inactive burrows within 100 m (IB100) were quantified within a circular plot of 100 m radius centered on the nest. Measurements (m) were obtained by extending a measuring tape 100 m from the nest in each of the four cardinal directions (N, S, E, W), allowing characterization of burrow density and activity within the immediate colony context. This scale reflects the structure and activity level of the Mexican prairie dog colony surrounding each nest site.
(d) Landscape scale: number of perches (NP), distance to perches (DP), distance to roads (DR), and distance to croplands (DC), variables associated with landscape heterogeneity and anthropogenic disturbance. Number of perches was recorded as a count, whereas distances to perches, roads, and croplands were measured in kilometers (km) using straight-line distances calculated in a Geographic Information System (GIS).

For each successful nest, a corresponding non-nest burrow was established to statistically evaluate which variables significantly influenced nest-site selection. Non-nest burrows were defined as inactive prairie dog burrows where no evidence of nesting activity (eggs, chicks, or adults attending a nest) was recorded during the breeding season. These non-nest burrows were located within the same colony and randomly selected in one of eight cardinal directions (N, NE, E, SE, S, SW, W, NW), at a minimum distance of 250 m from each successful nest. This design ensured spatial independence and avoided pseudoreplication. The same structural variables were measured at both nest and non-nest burrows across all four spatial scales. The 250 m distance threshold was selected because it approximates the radius of the immediate activity area of breeding Burrowing owls during the reproductive season, based on previous studies documenting local movements within this range [40,41,42].

### 2.3. Statistical Analysis

Nest-site selection was analyzed using a hierarchical, multiscale framework widely recommended for species responding to local- and landscape-level variables [1,43]. In a first step, exploratory binary logistic regression models (generalized linear models with a logit link and a binomial error distribution) were fitted separately for each spatial scale (burrow, site, colony, and landscape). The dependent variable was burrow status (TYPE: 1 = nest, 0 = non-nest). Non-nest burrows were selected within the same colony as each successful nest, controlling spatial structure and reducing potential pseudoreplication. Inference is therefore conditional on within-colony availability. Logistic regression was appropriate because it models the probability of a binary event as a function of structural predictors [44].

Prior to modeling, GLM assumptions were assessed by inspecting residuals, identifying influential observations (Cook’s distance), and evaluating model overfitting; no violations compromising model interpretation were detected. Subsequently, Spearman rank correlations (ρ) were calculated among continuous variables to identify strong associations and screen for potential multicollinearity. Predictor pairs with |ρ| ≥ 0.80 were considered highly collinear, and only one variable was retained based on ecological relevance and lower field measurement error. When correlations were moderate (0.70 ≤ |ρ| < 0.80), both variables were provisionally retained, provided that variance inflation factors (VIFs) in the fitted models indicated low residual collinearity (VIF < 3). This approach balances redundancy control with ecological interpretability [45,46].

Angular variables (orientation) were transformed into sine and cosine components (OR sin, OR cos) to allow their inclusion in linear models without loss of directional information. Using the reduced set of predictors, candidate models were fitted separately for each spatial scale. Model sets were generated using all biologically plausible combinations of predictors (a priori approach), following a multi-model inference framework. Model selection was based on the Akaike Information Criterion corrected for small sample sizes (AICc; [47]), which balances model fit and complexity and reduces overfitting in limited samples. For each model, the difference relative to the best-supported model (ΔAICc) and Akaike weights (Wi) were calculated, and models with ΔAICc ≤ 2 were considered competitive [48].

Predictor importance was evaluated primarily based on model support (AICc, ΔAICc, and Wi); additionally, 95% confidence intervals (95% CIs) for odds ratios were reported, and consistent statistical evidence was inferred when the 95% CI did not include 1. *p*-values were reported only as complementary information. Finally, predictors with the strongest statistical and ecological support at each spatial scale were integrated into a General Integrated Model, allowing joint evaluation of the relative contribution of micro- and macrohabitat factors to nest-site selection [49,50]. Prior to fitting the integrated model, residual collinearity among predictors was assessed using variance inflation factors (VIFs), confirming independence across spatial scales (all VIF < 3).

Model performance was evaluated using receiver operating characteristic (ROC) curves and the area under the curve (AUC), which quantifies the discriminatory ability of the models [51]. AUC values were calculated using the same dataset used for model fitting (internal AUC), as the objective of the analysis was comparative across spatial scales rather than predictive. Regression coefficients were interpreted as odds ratios (ORs) with 95% confidence intervals, allowing quantification of effect magnitude and direction [52]. All analyses were conducted in R (version 4.3.2; R Core Team, 2023) using RStudio (version 4.3.2), employing base functions from the stats package and additional tools for multi-model selection and model performance evaluation.

Because each successful nest was paired with a non-nest burrow within the same colony, the sampling design has a paired structure that could introduce dependence at the colony level. To assess the robustness of the results to potential clustering, sensitivity analyses were conducted using alternative model formulations, including generalized linear mixed models (GLMMs) with colony identity as a random intercept and conditional (paired) logistic regression with pair identity as a stratification factor. These alternative approaches yielded consistent effect directions and comparable effect sizes for the main predictors.

## 3. Results

During the 2010 and 2011 breeding seasons, a total of 56 breeding Burrowing owl nests were located, confirmed by the presence of nestlings or fledglings. In the first season, 37 nests were recorded: 27 in Nuevo León (Erial = 9, San Rafael = 9, and Soledad = 9) and 10 in San Luis Potosí (Manantial = 1 and Gallo = 9). In the second season, 19 nests were recorded: 13 in Nuevo León (Erial = 3, San Rafael = 5, and Soledad = 5) and 6 in San Luis Potosí (Manantial = 3 and Gallo = 3). All nests (100%) were located in burrows excavated by the Mexican prairie dog.

The model with the strongest support for nest-site selection at the burrow scale was mod2, which retained a comprehensive set of burrow structural attributes and immediate vegetation descriptors. A second competitive model (mod1) retained a reduced subset of these predictors, indicating that a smaller group of burrow and microhabitat variables captured much of the support observed at this scale. Together, these two models concentrated most of the empirical support at the burrow scale, with slightly greater support for mod2 than for mod1 (Table 1).

Across the two best-supported burrow-scale models, nest-site selection showed a consistent negative association with distance to the upper level (DUL) and a positive association with exit chamber length (ECL), whereas tunnel internal diameter and the remaining entrance and vegetation descriptors showed weaker or less consistent support across competing models (Table 2).

At the site scale, the best-supported model (mod14) retained distance to satellite burrows, vegetation height within 50 m, and vegetation cover within 50 m as predictors. A second competitive model (mod12) also retained satellite burrow presence (Table 1). In the top-ranked model, nest-site selection was negatively associated with distance to satellite burrows and vegetation height, whereas vegetation cover showed a positive association (Table 2).

At the colony scale, the most supported model (mod17) retained active and inactive burrows within 100 m as predictors, and a competitive alternative model (mod18) retained only active burrows (Table 1). Active burrows within 100 m showed strong support as a positive predictor of nest-site selection, whereas inactive burrows within 100 m showed weaker and less consistent support (Table 2).

At the landscape scale, the top-ranked model (mod20) retained number of perches, distance to perches, distance to roads, and distance to croplands as predictors (Table 1), and no alternative models received comparable support. Within this model, nest-site selection showed a negative association with number of perches and a positive association with distance to croplands, whereas distance to roads showed only weak support and distance to perches showed no clear association (Table 2).

Spearman’s rank correlation was used to screen for collinearity among continuous predictors prior to model fitting. Entrance diameter (ED) and tunnel internal diameter (TID) were highly correlated (ρ = 0.93), and only one of these variables was retained in multivariable models. Vegetation height within 50 m (VH50) and vegetation cover within 50 m (VC50) showed moderate correlation (ρ = 0.76); both variables were retained because the variance inflation factors in the selected models indicated low residual collinearity. No relevant residual collinearity was detected among predictors in the final models.

In the integrated general model, nest-site selection was negatively associated with entrance height, distance to satellite burrows, and number of perches, and positively associated with the density of active prairie dog burrows (Table 2). Other variables included in competing models showed limited support once multiple spatial scales were integrated.

The general integrated model with the strongest support was modG3, which retained entrance height, inactive burrows within 50 m, distance to satellite burrows, vegetation cover within 50 m, active burrows within 100 m, and number of perches (Table 1). A competitive alternative model (modG2) replaced vegetation cover with distance to upper level and included burrow height above ground (Table 1). Together, these two models accounted for nearly all empirical support for the integrated analysis (Table 1). In modG3, nest-site selection showed negative associations with entrance height, distance to satellite burrows, and number of perches, whereas active burrows within 100 m showed strong positive support (Table 2). In modG2, burrow height above ground showed a negative association, whereas distance to the upper level showed limited support (Table 2). In modG3, inactive burrows within 50 m and vegetation cover within 50 m showed no clear support once multiple spatial scales were integrated (Table 2).

Across spatial scales, the integrated general model received substantially more support than single-scale models, as reflected in its lower AICc and higher Akaike weight (Table 1). This pattern was further supported by model discrimination analyses, in which the general model performed well (AUC = 0.961), whereas single-scale models showed moderate (colony and landscape) to low (burrow and site) discriminatory power (Table 3).

## 4. Discussion

The results of this study indicate that nest-site selection by the Burrowing owl follows a hierarchical, multiscale process in which different sets of variables become relevant at each scale. Although all nests were located in burrows excavated by the Mexican prairie dog, the factors associated with burrow occupancy varied across scales, including burrow attributes, the spatial arrangement of burrows in the immediate surroundings, colony dynamics, and landscape characteristics. This integrated approach aligns with conceptual frameworks of multiscale habitat selection [1,31,43] and provides empirical evidence from the southernmost portion of the Chihuahuan Desert.

This pattern aligns with the sequential habitat-selection process described for the species in North American grassland systems, where nesting decisions emerge from hierarchical filters operating across spatial scales [30,32].

At the burrow scale, the predictors with the strongest support were distance to upper level (DUL; negative effect) and exit chamber length (ECL; positive effect), whereas entrance diameter (ED) showed a positive effect only in alternative models, suggesting a less consistent influence. These patterns indicate that Burrowing owls tend to occupy burrows with lower external exposure and greater internal development, which may reduce predation risk and contribute to greater microclimatic stability. Similar results have been documented in Black-tailed prairie dog colonies in the United States and Canada, where deeper and structurally more complex burrows were associated with higher nest occupancy and reproductive performance [53,54].

In contrast, entrance height (EH) showed a negative effect, consistent with a preference for more discreet burrow entrances. This pattern agrees with previous studies reporting positive selection for deeper and structurally complex burrows by Burrowing owls, which are associated with enhanced protection against predators and extreme environmental conditions [16,28].

At the site scale, owls had a higher probability of occupancy in areas closer to satellite burrows (distance to satellite burrows, DSB; negative effect with distance), with lower vegetation height within 50 m (VH50; negative effect) and greater vegetation cover within 50 m (VC50; positive effect). This pattern reflects a functional trade-off between openness and microrefuge availability, with relatively open spaces facilitating vigilance and predator detection, while intermediate vegetation cover may provide partial refuge. Importantly, this pattern does not imply selection for completely bare ground; rather, it supports selection against tall or dense vegetation while retaining low-to-intermediate cover that may provide microrefuge without compromising vigilance.

The avoidance of dense or tall vegetation likely reflects strong selection for habitats that enhance predator detection and reduce ambush opportunities, particularly in systems where avian and terrestrial predators exert significant pressure. In this context, the vigilance and alarm signaling of prairie dogs may offset the potential disadvantages of selecting burrows with lower entrance profiles.

Previous studies in the United States, particularly in the Mojave Desert, Nevada [30], and in Wyoming [29] have identified proximity to satellite burrows and vegetation structure as relevant factors in nest-site selection. Preference for low but not completely absent vegetation has been associated with improved predator detection efficiency and the availability of alternative refuges, supporting the role of microhabitat as an intermediate functional filter within the hierarchical selection process [29].

At the colony scale, the positive effect of active burrows within 100 m (AB100) and the marginal negative effect of inactive burrows within 100 m (IB100) suggest that Burrowing owls tend to occupy colonies with intermediate levels of Mexican prairie dog activity [18,19,20,21,30]. This association may reflect indirect benefits of prairie dog activity, such as alarm cues against predators, without implying a preference for highly saturated colonies.

This pattern supports the role of the Mexican prairie dog as an ecosystem engineer, whereby intermediate levels of activity maintain an open grassland structure without excessive microhabitat saturation, a condition consistent with higher Burrowing owl occupancy [14,15,16].

At the landscape scale, the predictors with the strongest support were number of perches (NP; negative effect) and distance to croplands (DC; positive effect). These results indicate that Burrowing owls tend to avoid landscapes with abundant perch structures, which may increase predation risk from larger raptors (e.g., *Buteo* spp., *Aquila chrysaetos*), and show a higher probability of occupancy in areas farther from croplands.

The negative influence of agricultural context on Burrowing owls has been documented previously, with surrounding land use affecting breeding pair density and reproductive output even when burrows are available [9,11,40]. The marginal effect of distance to roads observed here is consistent with previous reports of vulnerability to anthropogenic disturbance and associated mortality [28,55].

The integrated multiscale model showed the best overall performance, reinforcing the idea that nest-site selection arises from interactions among local- and landscape-level factors rather than from the influence of any single spatial scale. In this model, occupancy probability increased in burrows with lower entrance heights, in colonies with higher densities of active burrows (AB100), and in landscapes with fewer perches, while distance to satellite burrows (DSB) maintained a consistent negative effect. Variables such as inactive burrows within 50 m (IB50) and vegetation cover within 50 m (VC50) showed no statistical support once multiple scales were integrated, suggesting that their influence is secondary when the broader hierarchical context is considered.

This hierarchical pattern aligns with the framework proposed by [1] for selection across multiple orders. Our results align with previous multiscale analyses of Burrowing owls, which demonstrate that no single set of variables fully explains burrow occupancy; instead, nest-site selection arises from interactions among spatial scales [23,30].

Overall, these results suggest that Burrowing owls select nest sites through a hierarchical, multiscale filtering process that integrates burrow attributes, Mexican prairie dog colony dynamics, and the structure of the surrounding landscape. These findings underscore the importance of conserving both prairie dog colonies and the surrounding semi-natural grassland matrix, as habitat loss, agricultural expansion, and colony removal may disrupt habitat-selection processes in the southern Chihuahuan Desert.

The concordance between scale-specific models and the integrated general model reinforces the ecological robustness of the identified predictors and supports the interpretation that Burrowing owl nest-site selection responds to hierarchical processes rather than to isolated responses to individual variables.

## 5. Conclusions

This study demonstrates that nest-site selection by the Burrowing owl in the southern Chihuahuan Desert follows a hierarchical, multiscale process in which burrow occupancy arises from interactions among structural attributes of the burrow, activity within prairie dog colonies, and characteristics of the surrounding landscape. The clear predominance of the integrated general model over single-scale models underscores the need for analytical approaches that explicitly incorporate multiple spatial levels to adequately understand habitat-selection patterns. From a conservation perspective, these results underscore the importance of maintaining functional colonies of Mexican prairie dogs and grassland landscapes with low levels of anthropogenic disturbance, as alteration at any of these scales may compromise the availability of suitable breeding sites for the species in this region.

## Figures and Tables

**Table 1:** Model selection by spatial scale based on AICc. Entrance height (EH), entrance width (EW), entrance diameter (ED), burrow height above ground (BHG), distance to upper level (DUL), exit chamber length (ECL), tunnel internal diameter (TID), burrow orientation (OR), vegetation height within 1 m^2^ (VH1 × 1), vegetation cover within 1 m^2^ (VC1 × 1), active burrows within 50 m (AB50), inactive burrows within 50 m (IB50), satellite burrow presence (SB), distance to satellite burrows (DSB), vegetation height within 50 m (VH50), vegetation cover within 50 m (VC50), active burrows within 100 m (AB100), inactive burrows within 100 m (IB100), number of perches (NP), distance to perches (DP), distance to roads (DR), and distance to croplands (DC).

BURROW						
Model	Combination of variables	−2LL (Deviance)	k	AICc	∆AICc	wAICc
mod1	DUL + ECL + TID + OR + VH1 × 1 + VC1 × 1	131.07	7	145.07	0.31	0.35
mod2	EH + EW + ED + BHG + DUL + ECL + TID + OR + VH1 × 1 + VC1 × 1	122.76	11	144.76	0	0.408
mod3	BHG + DUL + ECL + TID + OR + VH1 × 1 + VC1 × 1	131.05	8	147.05	2.29	0.13
mod4	VH1 × 1 + VC1 × 1	147.34	3	153.34	8.58	0.006
mod5	EW + ED + BHG + DUL + ECL + TID + OR + VH1 × 1 + VC1 × 1	129.44	10	149.44	4.68	0.039
mod6	ED + BHG + DUL + ECL+ TID + OR + VH1 × 1+ VC1 × 1	130.52	9	148.52	3.76	0.062
mod7	TID + OR + VC1 × 1	149.76	4	157.76	13.00	0.001
mod8	OR + VH1 × 1 + VC1 × 1	147.30	4	155.30	10.54	0.002
mod9	ECL + TID + OR + VH1 × 1 + VC1 × 1	143.13	6	155.13	10.37	0.002
mod10	VC1 × 1	153.50	2	157.50	12.74	0.001
SITE						
Model	Combination of variables	−2LL (Deviance)	k	AICc	∆AICc	wAICc
mod11	AB50 + SB + DSB + VH50 + VC50	131.05	6	143.05	2.41	0.145
mod12	SB + DSB + VH50 + VC50	132.16	5	142.16	1.52	0.226
mod13	IB50 + SB + DSB + VH50 + VC50	131.05	6	143.05	2.41	0.145
mod14	DSB + VH50 + VC50	132.64	4	140.64	0	0.484
mod15	VC50	153.71	2	157.71	17.08	0
mod16	VH50 + VC50	148.59	3	154.59	13.96	0
COLONY						
Model	Combination of variables	−2LL (Deviance)	k	AICc	∆AICc	wAICc
mod17	AB100 + IB100	101.40	3	107.40	0	0.673
mod18	AB100	104.84	2	108.84	1.44	0.327
mod19	IB100	154.41	2	158.41	51.01	0
LANDSCAPE						
Model	Combination of variables	−2LL (Deviance)	k	AICc	∆AICc	wAICc
mod20	NP + DP + DR + DC	120.36	5	130.36	0	0.999
mod21	DP + DR + DC	139.35	3	145.35	14.99	0.001
mod22	DR + DC	142.28	2	146.28	15.92	0
mod23	DC + NP	142.28	2	146.28	15.92	0
mod24	DC	139.35	2	147.35	16.98	0
GENERAL MODEL						
Model	Combination of variables	−2LL (Deviance)	k	AICc	∆AICc	wAICc
modG3	EH + IB50 + DSB + VC50 + AB100 + NP	55.99	7	69.99	0	0.565
modG2	DUL + EH + IB50 + DSB + AB100 + NP	56.52	7	70.52	0.53	0.434
modG4	IB50 + DSB + VC50 + AB100 + NP	74.53	6	86.53	16.54	0
modG1	ED + DUL + VH1 × 1 + IB50 + DSB + VC50 + AB50 + NP	109.71	8	125.71	55.72	0

k indicates the number of parameters, ΔAICc represents the difference between each model and the best-supported model (lowest AICc), wAICc denotes Akaike weights.

**Table 2:** Effects of predictor variables in the best-supported models by spatial scale. Odds ratios (ORs) are derived from logistic regression models. Values < 1 indicate a negative association and values > 1 indicate a positive association with nest-site selection; CI indicates confidence interval. All predictor abbreviations are defined in Table 1 footnote and in the Section 2.

BURROW					
Model	Predictor	Odds ratio (OR)	Lower 95% CI	Upper 95% CI	*p*-value
mod2	EH	0.929	0.872	0.983	0.0144
mod2	EW	1.065	0.989	1.152	0.0974
mod2	ED	0.997	0.926	1.081	0.94
mod2	BHG	0.999	0.946	1.054	0.976
mod2	DUL	0.934	0.87	0.99	0.0416
mod2	ECL	1.044	1.008	1.086	0.0224
mod2	TID	1.033	0.959	1.11	0.368
mod2	OR	1	0.995	1.004	0.903
mod2	VH1 × 1	0.833	0.673	0.987	0.0576
mod2	VC1 × 1	0.988	0.958	1.016	0.403
mod1	DUL	0.922	0.866	0.969	0.00458
mod1	ECL	1.029	0.996	1.066	0.0929
mod1	TID	1.045	1.006	1.089	0.0288
mod1	OR	1	0.996	1.005	0.853
mod1	VH1 × 1	0.854	0.698	1.002	0.082
mod1	VC1 × 1	0.992	0.966	1.017	0.521
SITE					
Model	Predictor	Odds ratio (OR)	Lower 95% CI	Upper 95% CI	*p*-value
mod14	DSB	0.953	0.916	0.979	0.00319
mod14	VH50	0.784	0.63	0.956	0.021
mod14	VC50	1.033	1.007	1.064	0.0195
mod12	SB	1.047	0.917	1.207	0.492
mod12	DSB	0.951	0.91	0.978	0.00347
mod12	VH50	0.79	0.634	0.966	0.0269
mod12	VC50	1.032	1.006	1.063	0.0238
COLONY					
Model	Predictor	Odds ratio (OR)	Lower 95% CI	Upper 95% CI	*p*-value
mod17	AB100	1.194	1.122	1.292	6.54 × 10^−7^
mod17	IB100	0.804	0.662	1.012	0.0449
mod18	AB100	1.182	1.114	1.271	6.04 × 10^−7^
LANDSCAPE					
Model	Predictor	Odds ratio (OR)	Lower 95% CI	Upper 95% CI	*p*-value
mod20	NP	0.587	0.442	0.755	8.64 × 10^−5^
mod20	DP	0.999	0.991	1.008	0.886
mod20	DR	1.001	1	1.003	0.0714
mod20	DC	1.005	1.003	1.009	0.00056
GENERAL MODEL					
Model	Predictor	Odds ratio (OR)	Lower 95% CI	Upper 95% CI	*p*-value
modG3	EH	0.848	0.761	0.922	0.000592
modG3	IB50	1.13	0.9	1.437	0.292
modG3	DSB	0.937	0.892	0.97	0.00144
modG3	VC50	1.015	0.983	1.047	0.36
modG3	AB100	1.409	1.234	1.705	0.0000242
modG3	NP	0.507	0.316	0.751	0.00174
modG2	DUL	1.023	0.936	1.094	0.559
modG2	EH	0.842	0.746	0.927	0.00159
modG2	IB50	1.107	0.885	1.394	0.369
modG2	BHG	0.935	0.89	0.968	0.000986
modG2	AB100	1.41	1.233	1.704	0.0000243
modG2	NP	0.514	0.317	0.763	0.00252

**Table 3:** Discriminatory performance of the best-supported models by spatial scale based on ROC analysis. Area under the ROC curve (AUC) values indicate the ability of each model to discriminate between nest and non-nest burrows.

Spatial Scale	AUC	Lower 95% IC	Upper 95% CI
General Model	0.961	0.93	0.992
Colony	0.867	0.802	0.931
Landscape	0.798	0.718	0.879
Burrow	0.751	0.662	0.84
Site	0.587	0.481	0.694

## Data Availability

Data supporting the findings of this study are available from the corresponding author upon reasonable request.

## Abbreviations

The following abbreviations are used in this manuscript:

AB50	Active burrows within 50 m
AB100	Active burrows within 100 m
AICc	Akaike Information Criterion corrected for small sample sizes
AUC	Area under the ROC curve
BHG	Burrow height above ground
CI	Confidence interval
DC	Distance to croplands
DP	Distance to perches
DR	Distance to roads
DSB	Distance to satellite burrows
DUL	Distance to upper level
ECL	Exit chamber length
ED	Entrance diameter
EH	Entrance height
EW	Entrance width
GLM	Generalized linear model
GLMM	Generalized linear mixed model
IB50	Inactive burrows within 50 m
IB100	Inactive burrows within 100 m
NP	Number of perches
OR	Odds ratio
ROC	Receiver operating characteristic
SB	Satellite burrow presence
TID	Tunnel internal diameter
VC1 × 1	Vegetation cover within 1 m^2^
VC50	Vegetation cover within 50 m
VH1 × 1	Vegetation height within 1 m^2^
VH50	Vegetation height within 50 m
VIF	Variance inflation factor
Wi	Akaike weight
